# Research Progress on the Association Between GLP-1 Receptor Agonists and Cardiomyopathy

**DOI:** 10.31083/RCM37180

**Published:** 2025-08-30

**Authors:** Xiao Yang, Xinghui Li

**Affiliations:** ^1^The First Clinical Medical College, Gansu University of Traditional Chinese Medicine, 730000 Lanzhou, Gansu, China; ^2^Department of Cardiology, Gansu Provincial People's Hospital, 730000 Lanzhou, Gansu, China

**Keywords:** GLP-1RAs, cardiomyopathy, T2DM

## Abstract

Glucagon-like peptide-1 receptor agonists (GLP-1RAs) are a promising new class of drugs, whose clinical potential has recently been explored. Various preclinical studies and clinical trials initially demonstrated the efficacy of GLP-1RAs in treating type 2 diabetes mellitus (T2DM). However, long-term clinical practice has revealed that GLP-1RAs also exhibit significant efficacy and preventive effects in cardiovascular diseases. These effects are mediated through multiple gene pathways; thus, these drugs have shown substantial potential for further development in different clinical contexts. Cardiomyopathy, which constitutes a significant proportion of cardiovascular-related diseases, is increasingly prevalent, with its incidence rising annually. Thus, following the recent surge in research on cardiomyopathy, this review aims to summarize the latest findings regarding the association between GLP-1RAs and cardiomyopathy. This review begins with an introduction to GLP-1RAs, discussing their specific mechanisms of action. This article then addresses the pathogenesis, progression, and mechanisms of cardiomyopathy. Subsequently, a detailed analysis of the relationship between GLP-1RAs and cardiomyopathy is conducted. Finally, this review summarizes and discusses the latest literature on the impact of GLP-1RAs on the risk of various types of cardiomyopathy, as well as the potential underlying biological mechanisms, to provide clinical guidance on the use of GLP-1RAs in the treatment of cardiomyopathy.

## 1. Introduction

Patients with type 2 diabetes mellitus (T2DM) are at a high risk of developing cardiovascular diseases (CVDs) [1]. Cardiomyopathy, a group of heart diseases characterized by myocardial mechanical or electrical activity abnormalities, primarily includes hypertrophic, dilated, restrictive, and arrhythmogenic right ventricular cardiomyopathy. Among these, the incidence of diabetes-related cardiomyopathy has increased exponentially with the rising prevalence of diabetes [2]. Current treatment options, such as β-blockers and angiotensin receptor-neprilysin inhibitors (ARNIs), primarily focus on symptom relief but remain significantly limited in addressing the underlying causes of the disease [3]. Since 2005, glucagon-like peptide-1 receptor agonists (GLP-1RAs) have been used as key therapeutic agents for T2DM [4, 5, 6]. The 2021 European Society of Cardiology (ESC) guidelines recommend using GLP-1RAs as a glucose-lowering drug with cardiovascular protective effects for T2DM patients with CVDs or high cardiovascular risk [7].

Incretins are a group of gut hormones that promote postprandial glucose utilization and stimulate insulin secretion, with glucagon-like peptide-1 (GLP-1) being the most extensively studied incretin. GLP-1 is primarily synthesized by L cells in the lower gastrointestinal tract and also enhances β-cell function, providing beneficial effects across various tissues and organs [8]. GLP-1 stimulates insulin secretion and inhibits glucagon release, making it a novel compound for diabetes treatment. However, due to its rapid degradation by the enzyme dipeptidyl peptidase-4 (DPP-4) and its swift clearance by organs such as the kidneys (with a half-life of 1–2 minutes), GLP-1 cannot be used directly as a therapeutic agent [9]. Its receptor, glucagon-like peptide-1 receptor (GLP-1R), is expressed in various cell types, including monocytes/macrophages and smooth muscle cells, and mediates a range of biological effects, such as reducing neuroinflammation, suppressing appetite, and decreasing fat deposition [10]. The efficacy of GLP-1 varies significantly; while GLP-1 reduces blood glucose by stimulating insulin secretion, its short duration of action limits its effectiveness. In contrast, GLP-1RAs, compared to GLP-1, are more resistant to degradation, resulting in a more prolonged glucose-lowering effect [11].

Despite the extensive research on GLP-1RAs in blood glucose control and alleviation of diabetes complications, there remains a significant gap in research regarding their broader effects, especially concerning cardiovascular protection and other potential therapeutic benefits. Traditional medications often show limited efficacy in lipid lowering, blood sugar control, and blood pressure management. As a result, GLP-1RAs have become a focal point of intensive research. Compared with existing reviews, this paper presents two key innovations. First, it systematically establishes a novel classification framework (Types I–V) for new drugs based on the dual/multi-target agonist characteristics, providing a taxonomic basis for clinical drug development. Second, integrating multi-level mechanistic evidence from molecular, animal, and clinical studies reveals a coordinated regulatory network of GLP-1RAs in cardiomyopathy involving metabolism, inflammation, and structural remodeling. This review aims to explore the current understanding of the relationship between GLP-1RAs and cardiomyopathy, highlighting the latest advances in research and providing clinical insights for practical applications.

## 2. GLP-1RAs

**Fig. 1.**
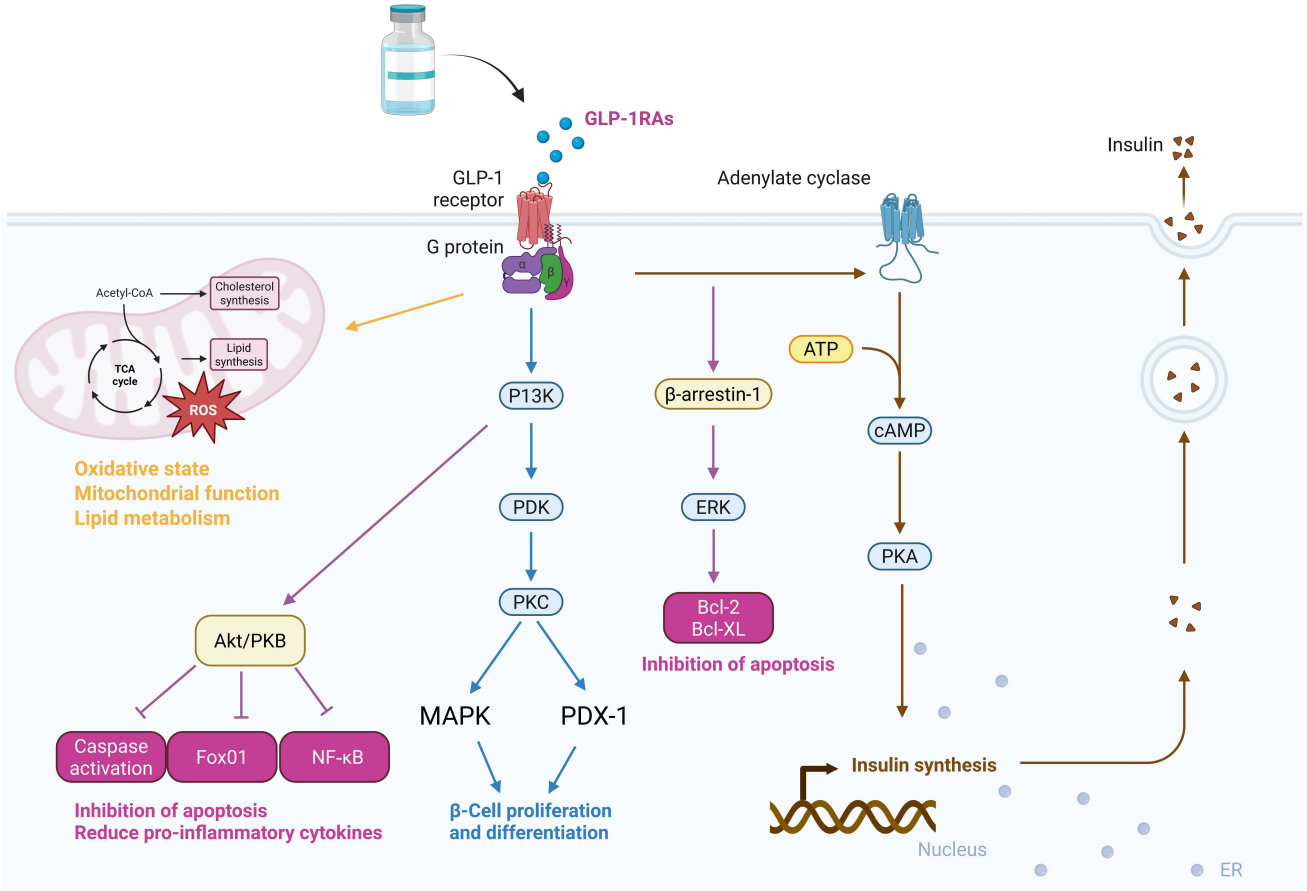
**Primary mechanisms of action and clinical regulatory effects of GLP-1RAs**. ROS, reactive oxygen species; PKB, protein kinase B; GLP-1RAs, glucagon-like peptide-1 receptor agonists; PI3K, phosphoinositide 3-kinase; PDK, phospholipid-dependent protein kinase‌; PKC, -‌protein kinase C‌; MAPK, mitogen-activated protein kinase; PDX-1, pancreatic duodenal homeobox-1‌; Bcl, B-cell lymphoma; cAMP, cyclic adenosine monophosphate; ERK, extracellular signal-regulated kinase; ATP, adenosine triphosphate; NF-κB, nuclear factor kappa B; GLP-1, glucagon-like peptide-1; PKA, protein kinase A. Created with Biorender.com.

GLP-1RAs exhibit different drug classifications, mechanisms of action, and regulatory effects, thus holding considerable potential for clinical application (Fig. 1).

### 2.1 Drug Classification

Currently, GLP-1RAs can be classified into two major categories based on market availability: (1) Approved drugs: liraglutide injection, semaglutide injection, and others; (2) Investigational drugs: These are primarily classified into five subtypes based on their target receptors: Type I targets GLP-1R, with representative drugs including oral semaglutide and benaglutide; Type II agents target both GLP-1R and gastric inhibitory polypeptide receptor (GIPR), exemplified by tirzepatide, which, as the first dual-receptor agonist, demonstrates significant advantages in weight reduction and metabolic regulation; Type III targets GLP-1R and Cagrilintide, with Cagrisema as a representative drug; Type IV targets GLP-1R and glucagon receptor (GCGR), with representatives including mazdutide and cotadutide; Type V targets GLP-1R and GIPR, with retatrutide as the representative drug [12].

GLP-1RAs are also classified into two categories based on their pharmacokinetic characteristics: (1) Short-acting drugs, such as exenatide and lixisenatide; (2) Long-acting drugs, including liraglutide, semaglutide, albiglutide, and dulaglutide. The primary pharmacodynamic difference between short-acting and long-acting GLP-1RAs is that short-acting agonists primarily reduce postprandial blood glucose by delaying gastric emptying, while long-acting agonists increase insulin secretion and inhibit glucagon production, leading to reductions in both postprandial and fasting blood glucose levels [13].

### 2.2 Mechanisms of Action

Recent research has elucidated several mechanisms by which GLP-1RAs exert cardiovascular protection, including effects on oxidative stress, inflammation, endoplasmic reticulum stress (ERS), apoptosis, and vascular/heart remodeling [14]. These mechanisms contribute to the overall physiological effects, primarily centered on glucose lowering, and position GLP-1RAs as drugs to improve endothelial damage and the progression of CVDs [15].

#### 2.2.1 Pathway-Related Mechanisms

The mechanistic pathways of GLP-1RAs can be integrated with clinical contexts to accelerate research progress. Stimulation of GLP-1R via G protein α-subunits increases adenylate cyclase activity, leading to increased adenosine triphosphate (ATP) production and elevated cyclic adenosine monophosphate (cAMP) levels, which in turn activate secondary pathways, including protein kinase A (PKA), phosphoinositide 3-kinase (PI3K), and mitogen-activated protein kinase (MAPK) signaling pathways [16]. GLP-1RAs counteract the effects of angiotensin II (ANG II), enhance its inactivation in the circulation, and affect target tissues such as glomerular endothelial cells and cardiomyocytes. They promote natriuretic effects and osmotic diuresis, inhibiting Na^+^/H^+^ exchanger NHE-3 (Na^+^/H^+^ exchanger-3‌), which is activated by ANG II [17]. Additionally, GLP-1RAs demonstrate significant anti-inflammatory effects by modulating immune responses, blocking nuclear factor kappa B (NF-κB) activation, and reducing the production of pro-inflammatory cytokines, making them highly valuable as therapeutic agents with broad clinical implications [18].

#### 2.2.2 Blood Glucose Regulation

Extensive research has explored the role of GLP-1RAs in glucose regulation. In type 1 diabetes mellitus (T1DM), some studies suggest that GLP-1RAs may delay the onset of T1DM by exerting potential immune-modulating and anti-inflammatory effects and protecting β-cells [19]. In T2DM, it has been demonstrated that edaglitazone, by activating GLP-1R, promotes insulin secretion and lowers blood glucose levels in mice, suggesting that it may be a potential GLP-1RA candidate for the prevention and treatment of metabolic diseases like T2DM [20]. In addition, prior treatment with GLP-1RAs in patients with T2DM significantly reduced mortality following ST-segment elevation myocardial infarction (STEMI) [21]. Triazinate (TZT), compared to a placebo, has been identified as the most effective GLP-1RA in controlling blood glucose in T2DM patients, as evidenced by reductions in HbA1c and fasting glucose concentrations [22]. Therefore, evaluating the changes in clinical indicators provides meaningful insights into assessing the efficacy of these drugs.

#### 2.2.3 Multifaceted Integration

Integrating molecular biology, animal experiments, and clinical trials has increasingly become a hot topic in recent research, aiding in clarifying the mechanisms of action. Berberich and Hegele [23] reported that blood lipid data collected as secondary outcomes in large clinical trials and smaller studies indicated that GLP-1RAs could modestly reduce low-density lipoprotein and total cholesterol levels, with most trials showing a slight reduction in fasting triglycerides. Additionally, Luna-Marco *et al*. [24] demonstrated through trials that GLP-1RAs treatment could improve oxidative status and mitochondrial respiratory function in T2DM patients, reduce leukocyte-endothelial interactions, and lower inflammation markers, thereby potentially reducing the risk of various diseases. Apart from objective indicator studies, research on the clinical significance of these indicators should not be overlooked. Kim *et al*. [25] analyzed brain samples from humans and mice and identified that GLP-1R neurons in the dorsomedial hypothalamus (DMH) are candidates for encoding pre-meal satiety. The intricate interactions between DMH^GLP-1R^ neurons and arcuate nucleus neuropeptide Y/agouti-related peptide (ARC^NPY/AgRP^) neurons regulate food intake, revealing previously unexplored hypothalamic mechanisms of GLP-1RAs in controlling pre-meal satiety, offering new neuro-targets for obesity and metabolic diseases.

It is evident that GLP-1RAs, as incretins that promote postprandial insulin secretion, exhibit glucose-lowering mechanisms that include increasing endogenous insulin secretion, reducing gluconeogenesis, inhibiting glucagon production by pancreatic α-cells, and decreasing β-cell apoptosis. Furthermore, they have been found to delay gastric emptying, promote weight loss, increase satiety, reduce blood pressure, improve dyslipidemia, alleviate inflammation, improve albuminuria, enhance cardiovascular function, and prevent thrombosis formation [26, 27].

### 2.3 Regulatory Efficacy

#### 2.3.1 Positive Regulation

Through mediation and meta-analysis, Scheen [28] highlighted that the improvement in blood glucose control has limited cardiovascular protective effects. However, the effects of GLP-1RAs were significantly greater than those of sodium-glucose cotransporter 2 inhibitors (SGLT2is). Tirzepatide, a dual agonist of GLP-1R and GIPR, demonstrated multidimensional improvements in cardiometabolic parameters in the SURPASS clinical trial series. These included reduced visceral fat mass (with an average decrease of 40%), improved insulin sensitivity, and significant alleviation of myocardial lipotoxicity [29, 30]. As shown in the LEADER trial, Liraglutide reduced the risk of major adverse cardiovascular events (MACE) by 13% in high cardiovascular-risk patients with T2DM. Its long-acting pharmacokinetic profile provides a basis for sustained cardiovascular protection [31]. TZT, a dual agonist of glucose-dependent insulinotropic polypeptide and GLP-1R has been shown to improve blood glucose levels and control body weight across various treatment regimens [29]. Subcutaneous injections of liraglutide (daily), semaglutide (weekly), dulaglutide, and efpeglenatide (weekly) all reduced the incidence of cardiovascular events. Moreover, weekly liraglutide, oral semaglutide, and exenatide also reduced mortality rates [5]. Therefore, exploring the dose-response relationship and its correlation with clinical disease treatment outcomes could clarify the optimal dosage and administration method. To date, randomized clinical trials of abiglutide, dulaglutide, liraglutide, and semaglutide have reported favorable cardiovascular prognoses [31].

#### 2.3.2 Negative Regulation

GLP-1RAs are commonly associated with gastrointestinal adverse effects, including nausea, vomiting, constipation, and diarrhea, with the highest incidence rates observed [32]. For example, studies have confirmed that liraglutide can cause gastrointestinal symptoms, making it the most common adverse event, with an incidence rate of 5% to 30% [33]. GLP-1RAs may delay gastric emptying, potentially increasing the risk of gastroesophageal reflux and aspiration [34]. To prevent such adverse effects, studies have identified factors such as age, gender, the number of concurrent oral medications, and a history of gastrointestinal diseases as risk factors for GLP-1RA-induced gastrointestinal side effects. A nomogram model has been developed to predict the risk of GLP-1RAs in clinical use, which is crucial for the safety and individualized administration of GLP-1RAs in T2DM patients [35]. This underscores the need for large-scale trials to define these drugs’ practical application and significance clearly.

## 3. Cardiomyopathy

### 3.1 Introduction to Cardiomyopathy

Cardiomyopathy is a heterogeneous group of diseases primarily characterized by abnormalities in myocardial mechanical or electrical activity. Its core features include structural abnormalities of the myocardium—such as ventricular hypertrophy, dilation, or fibrosis—and functional impairments involving either systolic or diastolic dysfunction [36]. According to the 2023 ESC consensus classification on cardiomyopathies, the global prevalence of cardiomyopathy is approximately 1 in 500, accounting for over 500,000 cardiovascular-related deaths annually [37]. Based on a recent nationwide claims database study in Japan, the clinically diagnosed prevalence of hypertrophic cardiomyopathy increased from 0.093% in 2017 to 0.111% in 2021. The highest prevalence was observed in older adults aged 85–89 years, with an estimated rate of 0.39% [38]. In comparison, the incidence of diabetes-related cardiomyopathy has markedly increased in parallel with the global rise in diabetes prevalence [39].

### 3.2 Classification and Clinical Phenotypes of Cardiomyopathy

Cardiomyopathies are broadly categorized into primary and secondary forms. Primary cardiomyopathies are mainly classified into five types based on structural and functional cardiac alterations: hypertrophic cardiomyopathy (HCM), dilated cardiomyopathy (DCM), non-dilated left ventricular cardiomyopathy, arrhythmogenic right ventricular cardiomyopathy (ARVC), and restrictive cardiomyopathy (RCM) [36]. Among them, HCM is characterized by asymmetric left ventricular wall thickening (≥15 mm), with approximately 50%–60% of cases associated with mutations in the *MYH7* or *MYBPC3* genes. Clinically, HCM presents with exertional dyspnea, chest pain, and a high risk of sudden cardiac death [40]. DCM is marked by ventricular dilation with impaired systolic function (left ventricular ejection fraction, LVEF <40%), and truncating mutations in the *TTN* gene are observed in 30–40% of cases. In later stages, DCM frequently leads to malignant ventricular arrhythmias [41]. RCM is primarily characterized by increased ventricular stiffness that results in diastolic dysfunction [42].

Secondary cardiomyopathies, on the other hand, arise as manifestations of systemic diseases. Common causes include infectious diseases (e.g., bacterial or viral infections), metabolic disorders (e.g., diabetic cardiomyopathy [DC], and amyloid-related myocardial changes), endocrine disorders (e.g., hyperthyroidism or hypothyroidism), connective tissue diseases (e.g., rheumatoid arthritis, systemic lupus erythematosus), ischemic heart diseases (e.g., coronary atherosclerosis, coronary vasospasm), hypersensitivity reactions (e.g., allergic responses to penicillin or sulfonamide drugs), and toxic injuries (e.g., myocardial damage caused by bacterial toxins in typhoid fever). DC is a diabetes-specific myocardial injury that occurs independently of coronary artery disease. Its characteristic pathological changes include myocardial microangiopathy, interstitial fibrosis, and lipid deposition [43]. Takotsubo cardiomyopathy, also known as stress-induced cardiomyopathy, is mainly characterized by acute and reversible left ventricular dysfunction, typically triggered by emotional or physical stress. The 90% of affected individuals are female [44].

### 3.3 Research on the Pathogenesis of Cardiomyopathy

#### 3.3.1 Vascular-Related Mechanisms

GLP-1RAs contribute to vascular health by mitigating endothelial dysfunction, a critical factor in the progression of atherosclerosis [45]. They enhance angiogenesis and suppress oxidative stress, thereby counteracting the downstream effects of endothelial damage. These effects include systemic inflammation, recruitment of monocytes, activation of pro-inflammatory macrophages, foam cell formation, proliferation of smooth muscle cells, and plaque development [46]. Mitochondrial dysfunction in cardiomyocytes represents a common pathological pathway shared by various types of cardiomyopathy. In HCM, mutations in sarcomeric proteins increase ATP hydrolysis demands, leading to energy depletion. In contrast, myocardial glucose utilization in patients with DCM decreases by 40–60%, resulting in a metabolic shift toward fatty acid oxidation for energy production [47]. Studies have shown that activation of the TGF-β/Smad3 signaling pathway promotes collagen deposition, increases myocardial stiffness, and drives fibrotic remodeling [48]. Furthermore, ferroptosis, triggered by inhibition of glutathione peroxidase 4 (GPX4), and programmed necrosis, mediated by the RIPK3–MLKL (receptor-interacting serine/threonine-protein kinase 3‌‌-mixed lineage kinase domain-like protein‌) pathway, play dominant roles in ischemic cardiomyopathy [49]. Experimental studies have demonstrated that antidiabetic agents, particularly GLP-1RAs (such as liraglutide) and SGLT2is (such as canagliflozin), exert significant cardioprotective effects against myocardial ischemia/reperfusion (I/R) injury in the context of diabetes. These effects are mediated through mechanisms including anti-necrotic actions and the activation of protective signaling pathways [50, 51]. These medications have demonstrated the ability to improve coronary blood flow, reduce acute thrombosis, alleviate I/R-related injury, limit infarct size, and prevent structural and functional remodeling in ischemic hearts. The underlying mechanisms involve inflammation suppression, oxidative stress reduction, and endothelial function restoration, all of which contribute to improved cardiac performance [52].

#### 3.3.2 Contributing Factors

Epicardial adipose tissue (EAT) substantially protects adjacent myocardium through its dynamic, brown adipose-like thermogenic function. However, it can also exert harmful effects by secreting pro-inflammatory and pro-fibrotic cytokines through paracrine or vascular secretion. EAT is a modifiable risk factor that can be evaluated using traditional and advanced imaging techniques [53]. The reduction of visceral fat is regarded as one of the non-glucose-lowering effects of GLP-1RAs, such as liraglutide [54], which may contribute to myocardial protection. GLP-1RAs have been shown to protect the heart from oxidative stress and reduce the expression of pro-inflammatory cytokines (interleukin (IL)-1β, TNF-α, IL-6, and monocyte chemoattractant protein-1 (MCP-1)) in the myocardium. Additionally, GLP-1RAs stimulate the inhibition of myocardial apoptosis, necroptosis, pyroptosis, and ferroptosis, enhancing autophagy and mitochondrial autophagy in the myocardium. These effects are mediated through the activation of exchange protein directly activated by cAMP (Epac) and the GLP-1R/PI3K/serine/threonine kinase B (Akt)/survivin pathway, downregulating reactive substance production. The protective actions of GLP-1RAs in the heart involve GLP-1R, kinases (protein kinase C epsilon (PKCε), PKA, Akt, adenosine 5^′^-monophosphate-activated protein kinase (AMPK), PI3K, extracellular signal-regulated kinase 1 and 2 (ERK1/2), mammalian target of rapamycin (mTOR), glycogen synthase kinase-3β (GSK-3β), protein kinase G (PKG), mitogen activated protein kinase 1 and 2 (MEK1/2), and mitogen activated protein kinase 3 (MKK3)), enzymes (heme oxygenase 1 (HO-1) and endothelial nitric oxide synthase (eNOS)), transcription factors (signal transducer and activator of transcription 3 (STAT3), cAMP-response element binding protein (CREB), nuclear factor (erythroid-derived 2)-like 2 (Nrf2), and forkhead box protein O3 (FoxO3)), adenosine triphosphate-sensitive potassium channel (KATP) channel opening, and mitochondrial permeability transition pore closure [49].

The etiology remains undetermined in approximately 30% of cardiomyopathy patients undergoing standard genetic testing. Emerging technologies such as proteomics and integrated multi-omics analyses are beginning to transform diagnostic approaches [55]. Existing pharmacologic therapies—such as β-blockers and ARNIs—primarily focus on symptom management and lack specific efficacy against pathogenic genes or molecular pathways. As a result, the 5-year mortality rate remains high, ranging from 20% to 50% [3]. Although some objective data have already supported the preventive effects of GLP-1RAs in cardiomyopathy, further research is needed to elucidate these mechanisms and optimize clinical application.

#### 3.3.3 The Role of GLP-1RAs in CVDs: Improvements in Left Ventricular Function and Clinical Outcomes

Among the contributing factors to cardiomyopathy, cardiovascular dysfunction often plays a central role in disease progression. In recent years, GLP-1RAs, initially developed for treating T2DM, have gained increasing attention for their cardioprotective effects—particularly in improving left ventricular function and reducing cardiovascular events. The cardiovascular benefits of GLP-1RAs extend beyond glycemic control and are thought to involve multiple mechanisms, including blood pressure regulation, reduced cardiac workload, and attenuation of myocardial injury.

To summarize the cardiovascular effects of GLP-1RAs—particularly about left ventricular function and cardiovascular outcomes—we systematically reviewed major clinical trials published in PubMed over the past five years. The inclusion criteria were: (1) the study population comprised of T2DM patients with established CVDs or at elevated cardiovascular risk; (2) the study evaluated the impact of GLP-1RAs on cardiovascular health, especially left ventricular function; and (3) the study reported explicit cardiovascular outcomes, such as major adverse MACE, left ventricular function, blood pressure, or myocardial performance. Based on these criteria, we identified 10 eligible clinical trials (Table 1, Ref. [29, 31, 43, 56, 57, 58, 59, 60]), which provide robust support for the cardiovascular applications of GLP-1RAs.

**Table 1.** **GLP-1RAs and cardiovascular disease studies**.

Study name	GLP-1RAs used	Population	Duration	Key findings on left ventricular function and clinical outcomes	Reason for CVDs relevance
LEADER trial [31]	Liraglutide	T2DM with high cardiovascular risk	3.5 years	13% reduction in major adverse cardiovascular events (MACE); improvement in left ventricular function through reduction in systemic inflammation	Significant reduction in MACE and cardiovascular risk factors
HARMONY Outcomes [56]	Albiglutide	T2DM patients with established CVDs	Median 1.6 years	Significantly reduced MACE; no direct assessment of left ventricular function (LVEF)	Included high-risk CVDs population; primary endpoint was cardiovascular outcomes (cardiovascular death, myocardial infarction, or stroke)
SURPASS series [29]	Tirzepatide	T2DM	12 months	Significant reduction in cardiovascular risk factors; improvements in myocardial work index and left ventricular strain in some subgroups	Investigates myocardial work index and left ventricular strain improvements
ORIGINS-RCE CardioLink-13 [57]	Semaglutide	T2DM with coronary artery disease (CAD)	6 months	Increased vascular progenitor cells; potential vascular repair mechanisms, reduction in granulocyte precursor cells	Investigates regenerative cardiovascular effects in CAD
CARMINE trial [58]	Dulaglutide	T2DM	6 months	Improvement in left ventricular longitudinal strain; reduction in arterial stiffness	Impact on left ventricular strain and arterial health
Tirzepatide study (JAMA) [43]	Tirzepatide	T2DM	28 weeks	Superior glycemic control, weight loss, improved left ventricular strain and myocardial function	Direct effect on myocardial function improvement and weight reduction
Ecosystem heart study [59]	Exenatide	T2DM with hypertension	12 weeks	Improvements in heart rate variability and systolic blood pressure, but no significant effect on left ventricular ejection fraction (LVEF)	Examines GLP-1RAs effect on heart rate variability and systolic BP in hypertension
SUMMIT CMR Substudy [60]	Tirzepatide	Obesity-related HFpEF	12 months	Reduced left ventricle mass and paracardiac adipose tissue	Demonstrated reversal of structural cardiac remodeling in HFpEF
Tirzepatide (SURPASS) [29]	Tirzepatide	T2DM	12 months	Reduction in cardiovascular risk factors; significant improvement in myocardial work index	Key outcome on myocardial work and heart function in T2DM
Tirzepatide [43]	Tirzepatide	T2DM	12 weeks	Greater improvement in left ventricular strain compared to other GLP-1RAs, reduction in HbA1c	Directly affects left ventricular strain and glycemic control

CVDs, cardiovascular diseases; T2DM, type 2 diabetes mellitus; MACE, major adverse cardiovascular events; BP, blood pressure; GLP-1RAs, glucagon-like peptide-1 receptor agonists; HbA1c, glycated hemoglobin A1c.

Several large-scale trials, including the LEADER and SURPASS series, have confirmed the positive cardiovascular effects of GLP-1RAs. For example, in the LEADER trial, liraglutide reduced the incidence of MACE by 13% in T2DM patients at high cardiovascular risk and improved left ventricular function by attenuating systemic inflammation [31]. The HARMONY Outcomes trial demonstrated that Albiglutide significantly reduced major adverse cardiovascular events in patients with T2DM and established CVDs, suggesting a potential role in cardioprotection [56]. Further evidence comes from studies on tirzepatide, which have demonstrated its ability to improve left ventricular strain and overall myocardial performance, particularly through favorable effects on cardiovascular risk factors and cardiac function [29, 43]. These findings indicate that GLP-1RAs improve glycemic control and exert direct benefits on myocardial function—especially left ventricular function—via a range of mechanisms. GLP-1RAs exhibit multifaceted roles in CVDs prevention and management by modulating metabolism, reducing inflammation, enhancing myocardial function, and improving microvascular health. These studies offer strong evidence for the clinical potential of GLP-1RAs in cardiovascular care.

## 4. GLP-1RAs and Cardiomyopathy

### 4.1 GLP1RAs and DC

#### 4.1.1 DC

DC is a chronic cardiovascular complication of diabetes, characterized by both structural and functional alterations in the myocardium, which can progress to heart failure (HF) and even mortality. A critical factor in the development of DC is mitochondrial dysfunction. Mitochondrial quality control mechanisms—including biogenesis, fusion, fission, and mitophagy—are essential for preserving mitochondrial integrity and normal physiological activity. In DC, these regulatory systems become disrupted, leading to insufficient mitochondrial fusion and excessive fission. This imbalance results in the accumulation of fragmented mitochondria within cardiomyocytes, contributing to cellular damage and impaired cardiac function. There is no targeted therapy or prevention strategy for DC, with blood glucose control remaining the primary management approach [43]. In insulin resistance or hyperinsulinemia conditions, DC manifests through a series of pathophysiological disturbances. These include impaired myocardial insulin signaling, mitochondrial dysfunction, ERS, disrupted calcium homeostasis, abnormal coronary microcirculation, overactivation of the sympathetic nervous system, dysregulation of the renin-angiotensin-aldosterone system, and maladaptive immune responses. Collectively, these factors lead to oxidative stress, myocardial fibrosis, hypertrophy, diastolic dysfunction, and eventually systolic HF [61].

#### 4.1.2 Related Studies and Mechanistic Analysis

4.1.2.1 Antioxidant Stress and Cell Apoptosis

Extensive studies have demonstrated that GLP-1RAs are critical in alleviating oxidative stress and apoptosis. Qian *et al*. [62] identified a novel oral GLP-1RAs, oxyntomodulin-derived hypoglycemic peptide 2 (OHP2). This compound reduced palmitate-induced oxidative stress and mitochondrial dysfunction by inhibiting intercellular lipid accumulation, showing promising potential for preventing and treating DC. These findings suggest that alterations in intercellular lipid levels help define the therapeutic indications and classification of OHP2. Yan *et al*. [47] confirmed that semaglutide alleviated oxidative stress and apoptosis in diabetic mice. It also improved cardiac function and reversed electrophysiological remodeling in DC mice. These effects were potentially mediated by activation of the SIRT1/AMPK signaling pathway and restoration of connexin 43 (Cx43) expression. In cellular models, Zhang *et al*. [63] showed that liraglutide mitigated high glucose (HG)-induced oxidative stress and apoptosis in cardiomyocytes. The anti-apoptotic effects were associated with downregulation of Bax, inhibition of caspase-3 activation, and upregulation of B-cell lymphoma-2 (Bcl-2) expression.

Meanwhile, one study by Chan *et al*. [64] indicated that glucose oxidation plays a key role in GLP-1RA-mediated attenuation of DC. These findings suggest that enhancing pyruvate dehydrogenase activity could represent a novel therapeutic strategy for DC. Zhu *et al*. [65] reported that combining ultrasound-targeted microbubble destruction (UTMD) with semaglutide-loaded PEGylated liposomes (Sem-PEG-lips) significantly reduced oxidative stress in DC. This effect was mediated through activation of the PI3K/Akt/Nrf2 signaling pathway and led to marked improvements in DC-related myocardial injury. Furthermore, Ji *et al*. [66] found that liraglutide exerted cardioprotective effects by inhibiting the inositol-requiring enzyme 1α (IRE1α)-mediated unfolded protein response (UPR) pathway and blocking C/EBP-homologous protein (CHOP)-mediated ERS-induced apoptosis. These results suggest that targeted modulation of specific molecular effectors within these signaling pathways may help elucidate the mechanisms of GLP-1RA action.

4.1.2.2 Other Mechanisms

GLP-1RAs play a significant role in regulating protein expression. Alobaid *et al*. [67] found that liraglutide enhances the expression of proteins in the integrin linked kinase (ILK)/PI3K/Akt/PTEN pathway by targeting GLP-1RAs, thereby exerting its cardioprotective effects in DC rats. Xue *et al*. [68] confirmed that liraglutide may improve myocardial injury in T2DM rats by dose-dependently inhibiting the expression of myocardial poly (adenosine diphosphate-ribose) Polymerase-1 (PARP-1).

GLP-1RAs improve cardiac health through multiple mechanisms, particularly in regulating arrhythmias. Previous studies have shown that GLP-1RAs exert cardioprotective effects by modulating intracellular signaling pathways, such as the ILK/PI3K/Akt/PTEN axis [67]. Xue *et al*. [69] found that GLP-1RAs alleviated structural cardiac damage in patients with T2DM, further supporting their potential role in arrhythmia management [70]. Ma *et al*. [71] demonstrated that liraglutide reversed HG-induced myocardial injury in cellular experiments. This effect was mediated through activation of the AMPK pathway and upregulation of GLP-1R expression. These findings highlight the importance of both protein expression levels and subcellular localization in determining the therapeutic efficacy of GLP-1RAs. In addition, the cardioprotective effects of GLP-1RAs appear to vary across patients with different body mass indices (BMI). Evidence suggests that improvements in cardiac structure are particularly pronounced in obese individuals. This may be attributed to the stronger impact of GLP-1RAs on ventricular structure and function in this population [45].

Moreover, GLP-1RAs, when used alone or in combination with other drugs, can also play a crucial role in preventing myocardial fibrosis. Trang *et al*. [72] demonstrated that empagliflozin and liraglutide treatment in diabetic rats reduced myocardial fibrosis and cell apoptosis. Empagliflozin modulates fatty acid and glucose metabolism, while liraglutide regulates inflammation and cell apoptosis in DC. Furthermore, Zhao *et al*. [73, 74] experimentally confirmed that liraglutide might offer cardioprotection by inhibiting P4hα-1-mediated myocardial fibrosis. Therefore, future research may focus on altering the “subtypes” of key targets within these established pathways to explore their unknown functions and develop gene-based treatments for refractory diseases like fibrosis. The references for the molecular mechanisms discussed above are summarized in Table 2 (Ref. [47, 62, 63, 64, 65, 66, 67, 68, 71, 72, 73, 74]).

**Table 2.** **The association between glucagon-like peptide-1 receptor agonists and diabetic cardiomyopathy (preclinical studies)**.

Category	Test ID & experimenter	Research subject	Experiment/Test method	Key indicators	Experiment/Test results
Antioxidative stress and anti-apoptosis	1. Qian *et al*. [62]	DC rats	High-fat diet and continuous streptozotocin injections for 8 weeks.	Cardiac function-related indicators.	OHP2 improved cardiac structure and function, reduced hyperlipidemia and myocardial lipid accumulation, and reversed oxidative stress and mitochondrial dysfunction in diabetic hearts.
	2. Yan *et al*. [47]	6-week-old male C57BL/6J DC mice	C57BL/6 mice were randomly divided into 4 groups: control group, semaglutide group, diabetes group, and diabetes + semaglutide treatment group. Type 1 diabetes was induced by intraperitoneal injection of streptozotocin. Mice in the semaglutide intervention group received subcutaneous injections of semaglutide (0.15 mg/kg) once weekly for 8 weeks.	Blood glucose levels, cardiac function, oxidative stress markers, apoptosis, and the expression of SIRT1, AMPK, and Cx43, as well as ECG changes.	Semaglutide treatment alleviated glucose metabolism disorders and improved cardiac dysfunction in diabetic mice. Additionally, semaglutide reduced oxidative stress and apoptosis in diabetic hearts, activated the SIRT1/AMPK pathway, and restored Cx43 expression, which had been reduced in diabetic mouse myocardium. ECG abnormalities, such as significantly prolonged RR, QRS, QT, and QTc intervals, were reversed following semaglutide treatment.
	3. Zhang *et al*. [63]	Sprague-Dawley neonatal rats	Cardiomyocytes from neonatal rats were cultured in Dulbecco’s Modified Eagle Medium.	Cell viability; Early apoptosis rate; SOD activity and MDA levels; Bax, Bcl-2, and cleaved/total CC3 protein levels.	Liraglutide effectively suppressed HG-induced early apoptosis and the increase in MDA levels, while significantly enhancing SOD activity. Additionally, liraglutide inhibited HG-induced upregulation of Bax and cleaved CC3 protein expression and promoted Bcl-2 expression.
	4. Chan *et al*. [64]	Male *Pdha1CM*^-⁣/-^ mice and αMHCCre mice	Experimental T2DM was induced by a 10-week high-fat diet supplemented with a single low-dose streptozotocin injection (75 mg/kg) at week 4. During the last 2.5 weeks, mice were randomly assigned to receive either vehicle control or liraglutide (30 µg/kg) treatment twice daily.	Cardiac function.	Liraglutide treatment improved glucose homeostasis in T2DM αMHCCre and *Pdha1CM*^-⁣/-^ mice, accompanied by mild weight loss. In αMHCCre mice, liraglutide alleviated diastolic dysfunction, as indicated by increased e’/a’ ratio and decreased E/e’ ratio, while systolic function parameters remained unaffected. Conversely, liraglutide failed to improve these diastolic function parameters in *Pdha1CM*^-⁣/-^ mice.
	5. Zhu *et al*. [65]	50 STZ-Induced diabetic rats	Fifty STZ-induced diabetic rats were randomly divided into the following groups: DC model group, Sem or Sem-PEG-lips single treatment groups, UTMD + Sem group, and UTMD + Sem-PEG-lips group (n = 10 each). Healthy rats served as the normal control group. The intervention lasted for 12 weeks.	BW and blood glucose levels. Myocardial injury and fibrosis. Antioxidant enzyme activity and expression levels of oxidative stress-related signaling pathway markers in myocardial tissue.	Compared to DC rats, the UTMD + Sem-PEG-lips group exhibited significantly higher BW and lower blood glucose levels. H&E and Masson staining showed substantial improvement in myocardial fibrosis and apoptosis in the combination treatment group. ELISA and western blot analysis further revealed significantly higher antioxidant enzyme activity and upregulation of PI3K/Akt/Nrf2 signaling pathway proteins in the combination-treated rats. These effects were reversed by PI3K inhibitor treatment.
	6. Ji *et al*. [66]	60 male Wistar rats	Over eight weeks, 40 rats were treated with intraperitoneal STZ for two weeks, while the remaining 20 rats were fed a normal diet and injected with an equivalent dose of citrate buffer. The rats were divided into three groups: non-DC group (control, n = 10), DC rats without liraglutide treatment (model, n = 14), and DC rats treated with liraglutide (100 µg/kg, n = 28).	Cardiac function. IRE-α, p-Perk, ATF6, and transcription factors. Apoptosis inducers CHOP, c-Jun N-terminal kinase, and caspase-12.	Liraglutide improved cardiac function in DC rats. IRE-α expression was significantly elevated in the DC group compared to controls, but liraglutide treatment reduced its expression. X-box binding protein-1 splicing was markedly increased in the model group but downregulated with liraglutide treatment. CHOP protein was upregulated in the DC group but was inactivated in the liraglutide-treated group.
Other mechanisms	7. Alobaid *et al*. [67]	24 Wistar albino rats	The rats were divided into four groups, with T2DM induced using a high-fat diet and STZ. Untreated control group rats were given 0.9% NaCl solution for 6 weeks, while the treatment group rats received 0.9% NaCl for 3 weeks, followed by subcutaneous liraglutide injections (150 µg/kg) for another 3 weeks.	Cardiac assessments included heart weight ratio, cardiac biomarkers (troponin I and creatine kinase-MB levels), antioxidant enzyme activities (glutathione peroxidase and superoxide dismutase), and MDA levels. ILK, P-PI3K, P-Akt, Bcl-2, CC3, Bax, and P-PTEN levels.	In the liraglutide-treated diabetic group, the heart weight ratio significantly decreased, and cardiac biomarkers (troponin I and creatine kinase-MB) improved. Antioxidant enzyme activities (glutathione peroxidase and superoxide dismutase) increased, while MDA levels decreased. Western blotting and immunohistochemistry revealed elevated levels of ILK, P-PI3K, P-Akt, and Bcl-2, as well as reduced levels of CC3, Bax, and P-PTEN, indicating reduced cardiomyocyte apoptosis.
	8. Xue *et al*. [68]	40 healthy 6-week-old male SD rats	The rats were randomly divided into a normal control group (n = 10) and a model group (n = 30), fed a standard diet and a high-sugar, high-fat diet, respectively. After successful modeling, the model group continued on the high-sugar, high-fat diet for 4 weeks and was further subdivided into a model group and an intervention group (split into high-dose and low-dose subgroups). The high-sugar, high-fat diet was maintained for 8 weeks, followed by drug intervention.	Fasting blood glucose and lipid levels. Whole heart tissues were dissected, weighed, and used to calculate the heart weight index. Myocardial pathological changes and cardiac PARP-1 expression.	The model group showed significantly higher body weight and heart weight index compared to the normal control group, while the intervention group exhibited a reduction in these indices, with more pronounced improvement in the high-dose subgroup. The model group displayed myocardial fiber disarray, inflammatory cell infiltration, and interstitial fibrosis. In the intervention group, myocardial pathology improved to varying degrees, with well-aligned myocardial fibers and clear striations; improvements were more pronounced in the high-dose subgroup. PARP-1 expression was significantly elevated in the myocardial tissue of the model group compared to the normal control group. Liraglutide intervention reduced PARP-1 expression in the myocardial tissue compared to the model group, with the high-dose subgroup showing a greater reduction, although levels remained higher than in the normal control group.
	9. Ma *et al*. [71]	Cardiomyocytes	Cardiomyocytes were subjected to high-glucose stress treatment.	Cell viability. Experiments were also performed to measure relevant indicators.	High-glucose treatment significantly increased inflammation and oxidative stress in cardiomyocytes, changes that were reversed by liraglutide. Exposure to high glucose reduced cell viability and increased apoptosis, but liraglutide treatment alleviated cardiomyocyte apoptosis. Furthermore, liraglutide activated the AMPK pathway and increased GLP-1R expression in response to treatment.
	10. Trang *et al*. [72]	Streptozotocin (65 mg/kg, intraperitoneal injection)-induced diabetic male Wistar rats	Diabetic rats were treated with empagliflozin (10 mg/kg/day, oral gavage) and/or liraglutide (200 µg/kg every 12 hours, subcutaneous injection) for 4 weeks.	Biochemical and echocardiographic evaluations were performed. Cardiac fibrosis, apoptosis, and the expression of metabolic and inflammatory signaling molecules in ventricular cardiomyocytes.	Empagliflozin and liraglutide normalized myocardial dysfunction in diabetic rats. Phosphorylation of acetyl-CoA carboxylase, carnitine palmitoyl transferase 1β, cluster of differentiation 36, and peroxisome proliferator-activated receptor-γ coactivator were upregulated, while glucose transporter 4 was downregulated. The ratio of phosphorylated AMP-activated protein kinase α2 to total AMPK α2 and the ratio of phosphorylated protein kinase B to total protein kinase B increased with empagliflozin or liraglutide treatment. Additionally, NLRP3, interleukin-1β, TNF-α, and cleaved caspase-1 were significantly downregulated in diabetic rats treated with empagliflozin and liraglutide.
	11. Zhao *et al*. [73, 74]	60 male Wistar rats	The rats were randomly divided into three groups: (1) Normal group (n = 20): Fed a standard diet. (2) Model group (n = 20): Fed a high-fat diet for 4 weeks, followed by an intraperitoneal injection of 30 mg/kg STZ. Rats were considered diabetic if FBG measured twice from the tail vein within 1 week after STZ injection exceeded 7.8 mM. (3) Liraglutide group (n = 20): Diabetic rats received subcutaneous liraglutide injections (0.09 mg/kg) for 16 consecutive weeks starting 1 week after STZ injection, while the normal and model groups received equivalent doses of saline.	At the end of the 16-week treatment with liraglutide or saline, BW, HW, HR, BP, electrocardiogram, and cardiac function were evaluated. Serum levels of TC, TG, LDL-C, NEFA, and hydroxyproline. Cardiac function was assessed through QRS wave, LVEDd, LVESd, and LVEF measurements. Myocardial fibrosis.	Compared to the model group, long-term liraglutide treatment reduced blood glucose levels and significantly alleviated lipid metabolism disorders. Liraglutide also improved impaired cardiac function. Furthermore, the improvement in cardiac dysfunction was associated with reduced myocardial fibrosis in diabetic hearts, as evidenced by decreased expression of P4hα-1, COL-1, COL-3, MMP-1, and MMP-9.

Note: AMPK, adenosine 5^′^-monophosphate-activated protein kinase; Bax, Bcl-2 associated x protein; Bcl-2, B-cell lymphoma-2; BP, blood pressure; BW, body weight; CC3, caspase-3; CHOP, C/EBP-homologous protein; COL, collagen; Cx43, connexin 43; DC, diabetic cardiomyopathy; ECG, electrocardiogram; ELISA, enzyme-linked immunosorbent assay; FBG, fasting blood glucose; GLP-1R, glucagon-like peptide 1 receptor; HG, high glucose; HR, heart rate; HW, heart weight; IRE-α, inositol-requiring enzyme α; LDL-C, low-density lipoprotein cholesterol; LVEDd, left ventricular end-diastolic diameter; LVEF, left ventricular ejection fraction; LVESd, left ventricular end-systolic diameter; MDA, malondialdehyde; MMP, mitochondrial membrane potential; NEFA, non-esterified fatty acids; NLRP3, nucleotide-binding oligomerization domain-like receptor family pyrin domain-containing 3; OHP2, oral hypoglycemic peptide 2; SOD, superoxide dismutase; STZ, streptozotocin; T2DM, type 2 diabetes mellitus; TC, total cholesterol; TG, triglyceride; TNF-α, tumor necrosis factor-α; UTMD, ultrasound-targeted microbubble destruction; αMHCCre mice, α-myosin heavy chain cre-expressing littermate mice; PI3K, phosphoinositide 3-kinase; Akt, serine/threonine kinase B; PEG, poly (ethylene glycol); ILK, integrin linked kinase; PTEN, phosphatase and tensin homolog deleted on chromosome ten; ATF6, activating transcription factor 6; SD, Sprague-Dawley; PARP-1, poly (adenosine diphosphate-ribose) polymerase-1.

**Fig. 2.**
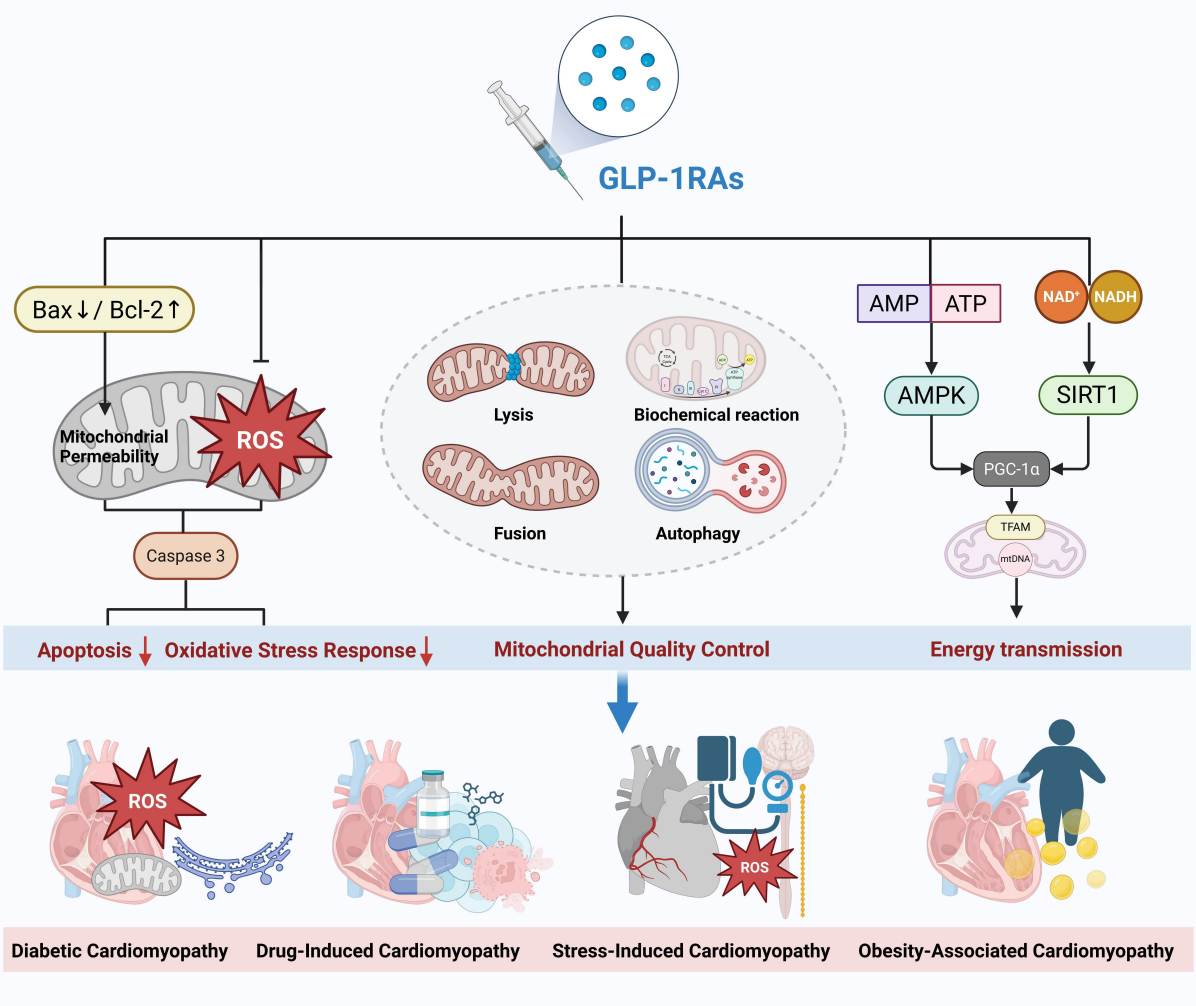
**Mechanisms of action and therapeutic effects of GLP-1 receptor agonists in different types of cardiomyopathy**. GLP-1RAs, glucagon-like peptide-1 receptor agonists; GLP-1, glucagon-like peptide-1; ROS, reactive oxygen species; AMP, adenosine monophosphate; ATP, denosine triphosphate; AMPK, adenosine 5^′^-monophosphate-activated protein kinase; Bax, Bcl-2 associated x protein; Bcl-2, B-cell lymphoma-2; TFAM, transcription factor a, mitochondrial; PGC-1α, peroxisome proliferator-activated receptor γ coactivator 1α; NADH, nicotinamide adenine dinucleotide. Created with Biorender.com.

Previous molecular studies have laid a theoretical foundation for understanding the role of GLP-1RAs in DC. However, many studies suggest that the cardioprotective effects of GLP-1RAs may extend beyond diabetes-related cardiomyopathy [62, 65]. They may also exert significant therapeutic benefits in other types of cardiomyopathies, such as drug-induced cardiomyopathy, stress-induced cardiomyopathy, and obesity-related cardiomyopathy. These findings indicate that the protective effects of GLP-1RAs may involve shared molecular mechanisms—such as the AMPK/SIRT1 signaling pathway—while also exhibiting disease-specific regulation. For instance, mitochondrial quality control plays a central role in DC (Fig. 2). The following sections will explore recent advances in research on GLP-1RAs across various types of cardiomyopathies, focusing on common and distinct molecular mechanisms.

### 4.2 GLP-1RAs and Other Types of Cardiomyopathy

#### 4.2.1 Non-Diabetic Cardiomyopathy (NDC)

In recent years, several pivotal clinical trials have provided important evidence supporting the use of GLP-1RAs in NDC. Findings from animal studies and preliminary exploratory research in patients with heart failure with preserved ejection fraction (HFpEF) suggest that GLP-1RAs may exert protective effects against obesity-related diastolic dysfunction by improving myocardial glucose metabolism, reducing lipid accumulation, and attenuating inflammation [75]. In the echocardiography substudy of the STEP-HFpEF Program, semaglutide demonstrated a potential to improve adverse cardiac remodeling compared with placebo, suggesting that semaglutide treatment may exert disease-modifying effects in patients with obesity-related HFpEF [76].

The SELECT trial (Semaglutide Effects on Cardiovascular Outcomes in People With Overweight or Obesity Who Do Not Have Diabetes, NCT03574597) was the first cardiovascular outcome study specifically targeting non-diabetic individuals with overweight or obesity (BMI ≥27 kg/m^2^ and established CVDs). The full results, published in 2023, revealed that 2.4 mg/week semaglutide treatment over 40 months reduced the risk of MACE—defined as cardiovascular death, non-fatal myocardial infarction, or stroke—by 20%. This effect was independent of baseline diabetes status; subgroup analysis showed a hazard ratio of 0.79 in non-diabetic participants [77]. Notably, in the SELECT trial, semaglutide significantly improved the 6-minute walk distance and Kansas City Cardiomyopathy Questionnaire (KCCQ) scores, suggesting a comprehensive enhancement of cardiac function and quality of life. Trend analyses of cardiac biomarkers indicated that semaglutide reduced levels of NT-proBNP and high-sensitivity cardiac troponin T (hs-cTnT), implying that its beneficial effects may be mediated through the attenuation of myocardial injury and improvement in ventricular remodeling (data derived from the biomarker sub-analysis of the SELECT trial) [77]. In the field of HFpEF, the STEP-HFpEF trial (2023) demonstrated that semaglutide improved KCCQ Clinical Summary Scores (KCCQ-CSS) by 16.6 points in non-diabetic HFpEF patients (‌New York Heart Association‌ (NYHA) class II–IV, LVEF ≥45%). It also significantly reduced body weight and C-reactive protein levels. Subgroup analyses indicated that improvements in cardiac structural parameters—including left ventricular mass index and E/e’ ratio—were consistent regardless of the presence of T2DM [78].

Current evidence suggests that the effects of GLP-1RAs on diabetes and NDC may differ dose-dependently. In DC, standard glucose-lowering doses (e.g., semaglutide 1.0 mg/week) are sufficient to confer cardioprotection. In contrast, higher doses (e.g., semaglutide 2.4 mg/week) may be required to achieve meaningful structural and functional improvements in NDC. This disparity may be related to differences in GLP-1R expression and the activation thresholds of downstream signaling pathways under varying pathological conditions. However, the precise mechanisms remain to be fully elucidated.

#### 4.2.2 Induced Cardiomyopathy

Doxorubicin (DXR)-induced cardiomyopathy is a severe health problem in cancer patients. Taşkıran *et al*. [79] showed that all three drugs tested, including oxytocin, liraglutide, and granulocyte colony-stimulating factor, alleviated DXR-induced cardiomyopathy in rat models. Additionally, Ussher *et al*. [80] investigated the adaptive response of GLP-1R to ventricular damage and its cardioprotective effects in mice. They found that GLP-1R knockout (*Glp1r*^-⁣/-^) mice did not show increased susceptibility to ischemia-induced mortality or experimental cardiomyopathy, suggesting that the systemic deletion of GLP-1R does not impair the adaptive response to ischemic or cardiomyopathic ventricular injury. Sepsis, a severe infectious disease, can lead to septic cardiomyopathy, which is fatal. Inflammation and oxidative stress are linked to the development of sepsis-induced cardiomyopathy. Wang *et al*. [81] demonstrated in an *in vitro* myocardial injury model that dulaglutide mitigates cell damage in lipopolysaccharide-induced cardiomyopathy by inhibiting inflammation and oxidative stress.

Li *et al*. [82] found in rat experiments that semaglutide protected against exercise-induced myocardial injury by activating the AMPK pathway, increasing autophagy, and reducing the production of reactive oxygen species (ROS) and inflammation-related proteins. Takotsubo syndrome (TTS), a stress-induced cardiomyopathy, is characterized by increased catecholamines, free radicals, inflammatory cytokines, endothelial dysfunction, and increased apoptosis. High-dose isoproterenol can be used in animal models to induce TTS-like myocardial injury. Bajic *et al*. [83] experimentally demonstrated that liraglutide protected myocardial cells from apoptosis in isoproterenol-induced TTS-like myocardial injury by downregulating the NF-κB pathway. Therefore, future studies should focus on exploring unknown pathways and their corresponding effects, thereby correlating cellular-level changes with genetic modifications to clarify the relationship between outcomes and functions, which could further facilitate clinical applications. The corresponding references for the induced cardiomyopathy models discussed above are summarized in Table 3 (Ref. [79, 80, 81, 82, 83, 84, 85, 86, 87]).

**Table 3.** **The association between glucagon-like peptide-1 receptor agonists and other types of cardiomyopathy (preclinical studies)**.

Category	Test ID & experimenter	Research subject	Experiment/Test method	Key indicators	Experiment/Test results
Induction types	1. Taşkıran *et al*. [79]	40 male Sprague-Dawley rats	A DXR-induced cardiomyopathy model was established in 32 rats. DXR was administered intraperitoneally every other day at a dose of 2.5 mg/kg/day for 6 doses. Eight rats served as the normal group without any treatment. The 32 DXR-treated rats were divided into four groups: (1) Placebo group: Received 0.9% NaCl saline solution via ip injection at 1 mL/kg/day. (2) Liraglutide group: Received liraglutide at 1.8 mg/kg/day via ip injection. (3) Oxytocin group: Received oxytocin at 160 µg/kg/day via ip injection. (4) G-CSF group: Received filgrastim (G-CSF) at 100 µg/kg/day via ip injection.	All treatments were administered for 15 days. On day 16, ECG were recorded under anesthesia. Blood samples were collected via tail vein puncture for biochemical analysis. Rats were then euthanized, and their hearts were harvested for immunohistochemical examination.	All three treatments alleviated the cardiotoxic effects of DXR on cardiac tissue, with the DXR + OX group showing the best results. The DXR + OX group exhibited the most preserved tissue integrity under light microscopy, the lowest CC3 immunoexpression, the highest QRS wave amplitude on ECG, and the lowest plasma levels of MDA, TNF-α, troponin T, and pro-BNP. OX treatment significantly reduced oxidative stress, apoptosis, and inflammation in the DXR-induced cardiomyopathy model, providing better tissue integrity, ECG parameters, and clinically relevant plasma biomarkers (troponin T and pro-BNP) compared to the other treatments. Liraglutide and G-CSF showed similar protective effects but were less pronounced than those of oxytocin.
	2. Ussher *et al*. [80]	10–12-week-old male *Glp1r*^-⁣/-^ mice and *Glp1r*^+⁣/+^ littermates, or 16–20-week-old *Glp1rCM*^-⁣/-^ mice and αMHC-Cre littermates	DXR-induced cardiomyopathy was established through a single intraperitoneal injection of DXR (20 mg/kg) in *Glp1r*^-⁣/-^ mice, *Glp1r*^+⁣/+^ littermates, or C57BL/6J mice. Mice were followed up for 10 days.	Cardiac tissues from surviving mice were analyzed histologically or for gene and protein expression. HR was assessed via telemetry, plasma ANP and insulin and further molecular.	*Glp1r*^-⁣/-^ hearts exhibited chamber-specific differences in gene expression but showed normal mortality and LV remodeling following MI or experimental doxorubicin-induced cardiomyopathy. GLP-1RAs like liraglutide demonstrated strong cardioprotective effects and improved survival rates in *Glp1rCM*^-⁣/-^ mice even after LAD coronary artery ligation. Although liraglutide increased HR in *Glp1rCM*^-⁣/-^ mice, these mice exhibited significantly lower baseline HR compared to controls.
	3. Wang *et al*. [81]	*In Vitro* cardiomyocyte injury model	Cells were stimulated with dulaglutide at concentrations of 5, 10, 50, 100, 500, and 1000 nM for 24 hours. Additionally, cells were incubated with LPS (1 µg/mL) with or without dulaglutide (50 and 100 nM) for 24 hours.	Expression of NOX-1 and iNOS. TNF-α, IL-1β, IL-17, MMP-2, and MMP-9 in H9c2 cardiomyocytes. NO production. Levels of CK-MB and cTnI. TLR4, MyD88, and p-NF-κB p65 in the nuclei of H9c2 cardiomyocytes.	Dulaglutide improved LPS-induced oxidative stress by inhibiting mitochondrial ROS production, increasing reduced GSH levels, and downregulating NOX-1. Dulaglutide reduced LPS-induced cardiomyocyte injury by downregulating CK-MB and cTnI, inhibiting iNOS expression, and reducing NO production. Dulaglutide significantly reversed LPS-induced production of inflammatory cytokines and upregulation of MMPs by inhibiting the TLR4/MyD88/NF-κB signaling pathway.
	4. Li *et al*. [82]	LPS-Induced H9c2 cells	A sedentary control group, an overtraining group, and an overtraining group treated with semaglutide underwent progressive swim training for 10 consecutive weeks.	The effects of semaglutide on LPS-induced oxidative stress injury and inflammatory responses in H9c2 cells. After the last training session, body weight, myocardial morphological changes, injury markers, and inflammation-related protein expression were analyzed in the model rats.	In LPS-treated H9c2 cells, semaglutide at three concentrations significantly increased cell survival rates and inhibited cardiomyocyte apoptosis. Additionally, semaglutide activated the AMPK pathway, improved autophagy, and suppressed ROS production in LPS-treated H9c2 cells. Long-term semaglutide treatment significantly reduced myocardial injury markers. Histopathological analysis showed that semaglutide improved myocardial morphological changes, reduced lipid accumulation areas, and markedly decreased the expression levels of NF-κB, TNF-α, and IL-1β.
	5. Bajic *et al*. [83]	Male Wistar rats	The rats were divided into four groups: Control group (C), n = 6: Treated with 1 mL/kg saline subcutaneously (sc) for 10 days, followed by 1 mL/kg saline sc on days 9 and 10. Liraglutide group (L), n = 6: Treated with 1.8 mg/kg liraglutide sc daily from day 1 to day 10, followed by 1 mL/kg saline sc on days 9 and 10. Isoproterenol group (I), n = 8: Treated with 1 mL/kg saline sc for 10 days, followed by 85 mg/kg isoproterenol sc on days 9 and 10 to induce myocardial injury. Liraglutide + Isoproterenol group (L + I), n = 9: Treated with 1.8 mg/kg liraglutide sc daily for 10 days, followed by 85 mg/kg isoproterenol sc on days 9 and 10.	On day 11, rats were euthanized, and their hearts were collected for histopathological and immunohistochemical analyses.	Liraglutide reduced isoproterenol-induced cardiomyocyte apoptosis by decreasing cleaved CC3, Bax, and NF-κB expression, while increasing Bcl-2 expression. In isoproterenol-treated rats, increased NF-κB was positively correlated with pro-apoptotic markers (Bax and CC3) and negatively correlated with the anti-apoptotic marker Bcl-2. Liraglutide treatment increased Bcl-2 and reduced NF-κB, Bax, and CC3 levels, maintaining the same correlations between NF-κB and apoptosis markers as in the isoproterenol-only group.
Other types	6. Shiraki *et al*. [84]	Spontaneous DCM in non-diabetic J2N-k hamsters. Male cardiomyopathic J2N-k hamsters (n = 8) and normal J2N-n hamsters (n = 64) were studied.	J2N-k hamsters were treated with PBS (HF group), low-dose liraglutide (HF-L group), or high-dose liraglutide (HF-H group).	Echocardiography was performed, followed by left ventricular catheterization to measure LV pressure. Blood samples were collected for HbA1c measurement. ATP and FFA quantification, collagen content measurement in cardiac tissue, and respiration analysis were also conducted.	In failing hearts, GLP-1 analogs further deteriorated cardiac function, with myocardial protein expression indicating an energy-deficient state. Indirect calorimetry showed that failing hearts consumed more energy and carbohydrates compared to normal hearts; GLP-1 analog administration exacerbated this trend. In a supplemental experiment, the HF-H group was provided with a 10% glucose solution. This intervention significantly improved cardiac function and reduced fibrosis while further increasing carbohydrate utilization and reducing lipid utilization. Prognosis in the HF-H-G group was also significantly improved.
	7. Vyas *et al*. [85]	Mouse model of DCM (TG9)	From day 56 after birth, GLP-1 agonist exenatide was administered twice daily to a DCM (TG9) mouse model. TG9 mice predictably develop congestive HF and secondary insulin resistance, with mortality occurring at 12 weeks of age.	Serum analysis, myocardial glucose uptake, protein expression, brain natriuretic peptide (BNP) RNA quantification, and echocardiography were performed.	Glucose homeostasis was evaluated by measuring glucose tolerance at 8 and 10 weeks and tissue 2-deoxyglucose uptake at day 75. Compared to vehicle-treated TG9 mice, exenatide treatment improved glucose tolerance, myocardial GLUT4 expression, 2-deoxyglucose uptake, cardiac contractility, and survival rates. Exenatide also increased phosphorylation of AMP kinase and Akt. Additionally, exenatide mitigated the adverse effects of the GLUT4 antagonist ritonavir on TG9 mouse survival.
	8. El-Kharashi *et al*. [86]	Wistar rats with Cirrhosis	Rats were divided into four groups: control group, EXA group, TAA group, and TAA + EXA group.	AST, ALT, FBG, and troponin I levels. Cardiac HOTAIR and SIRT1, as well as GLP-1R in the liver and heart.	EXA administration in control rats did not produce significant changes. TAA induced cirrhosis with notable changes in insulin resistance and cardiac function. GLP-1R, HOTAIR, and SIRT1 expression in cardiac tissue were significantly reduced, while troponin I levels were markedly elevated. The TAA + EXA group showed recovery of liver structure and function. EXA treatment significantly improved cardiac parameters, which were associated with increased expression of cardiac GLP-1R and HOTAIR.
	9. Sukumaran *et al*. [87]	Zucker rats with high-salt diet (6% NaCl)	Eight-week-old lean (^+⁣/+^) and obese (fa/fa) Zucker rats were treated with either vehicle or liraglutide (0.1 mg/kg/day, sc) for 8 weeks.	SBP. Myocardial function. Coronary vascular function was assessed *in vivo* in anesthetized rats. Myocardial gene expression and protein levels of vasoactive factors, inflammatory markers, oxidative stress markers, and remodeling biomarkers.	Compared to vehicle-treated fa/fa rats, liraglutide treatment significantly improved acetylcholine-mediated vasodilation in arterioles and small arteries. Liraglutide downregulated NOX-1 mRNA and reduced ET-1 protein expression. Additionally, liraglutide significantly decreased the expression of pro-inflammatory and pro-fibrotic biomarkers (NF-κB, CD68, IL-1β, TGF-β1, and osteopontin) and nitrotyrosine in fa/fa rats. However, liraglutide did not significantly reduce perivascular fibrosis.

Note: Akt, serine/threonine kinase B; ALT, alanine aminotransferase‌; AMP, adenosine monophosphate‌; AMPK, adenosine 5^′^-monophosphate-activated protein kinase‌; ANP, atrial natriuretic peptide; AST, aspartate aminotransferase‌; ATP, adenosine triphosphate; Bax, Bcl-2-associated X protein; Bcl-2, B-cell lymphoma/leukemia-2; CC3, caspase-3; CK-MB, creatine kinase-MB; cTnI, cardiac troponin I; DCM, dilated cardiomyopathy; DXR, doxorubicin; ECG, electrocardiogram; ELISA, enzyme-linked immunosorbent assay; EXA, exenatide; FBG, fasting blood glucose; FFA, free fatty acid; G-CSF, granulocyte colony-stimulating factor‌; GLP-1, glucagon-like peptide-1; GLP-1R, glucagon-like peptide-1 receptor; GLP-1RAs, glucagon-like peptide-1 receptor agonists‌; GSH, glutathione‌; HF, heart failure; HR, heart rate; IL, interleukin; iNOS, inducible nitric oxide synthase; LAD, left anterior descending artery; LPS, lipopolysaccharide; LV, left ventricular; MDA, malondialdehyde‌; MI, myocardial infarction; MMP, mitochondrial membrane potential‌; NF-κB, nuclear factor kappa B‌; NO, nitric oxide; NOX-1, NADPH oxidase-1‌; OX, oxytocin‌; ROS, reactive oxygen species‌; SBP, systolic blood pressure; TAA, thioacetamide; TNF-α, tumor necrosis factor-α; HOTAIR, HOX transcript antisense RNA; ET, endothelin‌; HbA1c, glycated hemoglobin A1c; TLR4, -‌Toll-like receptor 4‌; PBS, phosphate buffered saline‌.

#### 4.2.3 Other Types of Cardiomyopathy

DCM is characterized by the enlargement of the left ventricle or both ventricles, accompanied by systolic dysfunction. Shiraki *et al*. [84] studied non-diabetic J2N-k hamsters with spontaneous DCM and concluded that the failing myocardium is relatively deficient in glucose as an energy source, which may limit ATP synthesis and lead to deterioration of cardiac function. In the non-diabetic DCM model, liraglutide-induced energy starvation may exacerbate heart failure. This mechanism involves limiting the supply of glucose, reducing ATP synthesis, thereby leading to cardiac function deterioration. Therefore, when administering incretin-based therapies to HF patients, carefully considering adequate energy supply and carbohydrate intake is essential. Additionally, Vyas *et al*. [85] found that exenatide improved glucose homeostasis and prolonged the survival of DCM mice (TG9 model). This suggests that incretin-based therapies enhance myocardial glucose uptake in DCM.

Cirrhosis can impair liver function and potentially affect cardiac function due to disruptions in product generation. El-Kharashi *et al*. [86] concluded that exenatide promoted the cardioprotective effects of HOX transcript antisense RNA (HOTAIR) in a rat model of cirrhosis, offering a potential new therapeutic strategy for cirrhotic cardiomyopathy. Obesity is a significant independent risk factor for CVDs, and chronic obesity can lead to cardiac dysfunction, progressing to obesity-related cardiomyopathy. Therefore, there is a need to explore safer and more effective treatments for obesity-related cardiomyopathy. Sukumaran *et al*. [87] found that chronic liraglutide treatment improved nitric oxide (NO)-mediated vasodilation in both the coronary arteries and microcirculation, partially normalizing myocardial remodeling independent of body weight or blood glucose changes. This suggests that improving microvascular perfusion could enhance oxygen and nutrient supply to the myocardium, providing beneficial effects on preventing and treating obesity-related cardiomyopathy.

## 5. Conclusion

In recent years, multiple studies have confirmed that GLP-1RAs exhibit distinct therapeutic advantages in cardiomyopathy through various mechanisms, including regulation of metabolic reprogramming, suppression of inflammatory cascades, and improvement of myocardial remodeling. This review is the first to classify GLP-1RAs into five novel subtypes (Types I–V) based on their receptor-targeting profiles, thereby overcoming the limitations of traditional classifications based on drug half-life. Additionally, we constructed a multiscale (molecular–cellular–systemic) interactive model that elucidates the multilayered associations between GLP-1RAs and cardiomyopathy, revealing their core mechanisms in improving myocardial conditions through energy metabolism reprogramming, stabilization of the inflammatory microenvironment, and reversal of myocardial fibrosis. These findings provide a theoretical foundation for the development of precision treatment strategies.

GLP-1RAs play a pivotal regulatory role in DC by targeting key pathological pathways. Specifically, they improve mitochondrial dysfunction by activating the SIRT1/AMPK signaling pathway, which enhances mitophagy and restores mitochondrial fusion/fission balance in diabetic myocardium [47]; they alleviate oxidative stress by activating the PI3K/Akt/Nrf2 pathway, thereby reducing ROS production and myocardial lipid peroxidation [65]; and they inhibit apoptosis by blocking the IRE1α–CHOP-mediated ERS pathway, downregulating Bax and caspase-3, and upregulating Bcl-2 expression [63]. Moreover, GLP-1RAs exhibit dose-dependent cardioprotective effects across different types of cardiomyopathy. In HCM, they reduce collagen deposition and ventricular stiffness via suppression of the TGF-β/Smad3 pathway [48]; and in catecholamine-induced cardiomyopathy, they mitigate myocardial apoptosis through downregulation of the NF-κB signaling pathway [83].

Tuttle *et al*. [88] confirmed that weekly semaglutide administration reduced the risk of kidney disease endpoints and improved risk categorization. Several studies have shown that GLP-1RAs not only contribute to weight loss but also reduce CVD’s risk factors such as blood pressure and blood lipids, with consistent beneficial outcomes observed across various subtypes [89, 90, 91]. A meta-analysis of 22 studies demonstrated that GLP-1RAs can lower the risk of MACE [92]. Therefore, future studies with larger sample sizes are needed to validate and expand the preventive role of GLP-1RAs, while further elucidating the mechanisms of multi-cellular functional regulation.

In addition, Simanenkova *et al*. [93] found through experimentation that GLP-1RAs exerted neuroprotective effects on T2DM rats by directly influencing neuronal survival. Tabernacki *et al*. [3] demonstrated that GLP-1RAs might be the preferred treatment for T2DM while simultaneously reducing the risk of lung cancer. With the growing body of basic and clinical research, a relationship between GLP-1RAs and various cancers, including medullary thyroid carcinoma, pancreatic cancer, colon cancer, prostate cancer, breast cancer, cervical cancer, endometrial cancer, and ovarian cancer, has been observed [94]. This underscores the need for further exploration of GLP-1RA’s multi-systemic therapeutic potential.

GLP-1RAs have attracted significant attention across multiple research fields due to their strong clinical potential. The development of novel GLP-1RAs has opened new therapeutic avenues for cardiomyopathy. Dual-receptor agonists such as tirzepatide demonstrate pleiotropic advantages by synergistically modulating GIPR and GLP-1R signaling pathways, leading to improvements in myocardial metabolism, suppression of inflammation, and reversal of fibrosis [88]. The long-acting profile of liraglutide and its well-established cardiovascular protective effects make it a preferred therapeutic option for patients with diabetes and coexisting cardiomyopathy [88].

At the same time, several studies have pointed out the disadvantages of GLP-1RAs. For example, Rodriguez-Valadez *et al*. [95] analyzed a systematic review that included 9 GLP-1RA’s trials and 13 SGLT2i trials, confirming that the reduction in HbA1c by GLP-1RAs may increase the risk of MACE. Moreover, compared with DPP-4 inhibitors and SGLT2 inhibitors, GLP-1RAs significantly reduced hospitalization rates in patients with HF [96]. This highlights the need further to explore the relevant fields in a multi-system context. Adverse effects of GLP-1RAs are almost unavoidable. Studies have shown that semaglutide, exenatide, liraglutide, and dulaglutide are all associated with an increased risk of gastrointestinal side effects, including headache, nausea, vomiting, abdominal pain, and diarrhea [97]. Therefore, improving patient adherence to these medications remains a critical challenge. Future research could aim to develop “positive-side-effect” and “negative-side-effect” drugs to neutralize adverse effects, thus alleviating patient discomfort during treatment. In addition, a comparison of the specific side effects of different drugs in various clinical contexts could help identify whether combination therapies might reduce side effects.

In addition, several critical bottlenecks hinder translational progress from basic to clinical research. First, there are limitations in current model systems: approximately 85% of mechanistic studies rely on rodent models, such as diabetic mice or drug-induced cardiomyopathy in rats. However, the pathophysiology of human cardiomyopathy is more heterogeneous. For example, patients with DC often present with complex comorbidities including microvascular dysfunction, autonomic neuropathy, and atherosclerosis, which are difficult to replicate in existing animal models fully [47]. Second, substantial interspecies biological differences exist. The dose-response relationships observed in clinical settings differ significantly from those in animal studies. For instance, NDC requires higher doses (e.g., 2.4 mg/week semaglutide) for structural improvement. This discrepancy may be due to species-specific differences in GLP-1R expression (GLP-1R density in human cardiomyocytes is 40–60% lower than in mice), receptor signaling efficiency (e.g., humans have a higher cAMP production threshold), and drug tissue distribution [88]. Third, human pathological heterogeneity remains a major challenge. Most clinical trials rely on composite endpoints and lack mechanistic stratification for specific cardiomyopathy subtypes (e.g., obesity-related vs. diabetes-related forms). Recent single-cell sequencing data indicate significant individual variability in GLP-1R-positive myocardial cell proportions (ranging from 3% to 22%), suggesting the need for biomarker-guided precision treatment strategies [55].

From a translational perspective, combination therapy strategies involving GLP-1RAs warrant special attention. Preclinical studies have demonstrated that liraglutide combined with SGLT2 inhibitors can attenuate myocardial fibrosis through dual regulation of metabolic and inflammatory pathways [72]. Moreover, co-administration of GLP-1RAs and SGLT2 inhibitors significantly reduces the incidence of gastrointestinal adverse effects, possibly due to synergistic modulation of gut-brain axis signaling [98]. In clinical practice, for patients with HFpEF, combining GLP-1RAs with ARNIs may improve ventricular compliance through complementary mechanisms: GLP-1RAs primarily enhance myocardial energy metabolism and microcirculatory perfusion, while ARNIs target myocardial fibrosis and reduce ventricular wall stress. GLP-1RAs combined with adipokine-targeting agents (e.g., leptin receptor agonists) may achieve more pronounced cardiac functional improvement through dual mechanisms involving central appetite regulation and peripheral lipid metabolism in patients with obesity-related cardiomyopathy.

Importantly, three context-specific challenges must be addressed for the effective clinical use of GLP-1RAs: (1) in patients with acute decompensated cardiomyopathy, the impact of gastrointestinal side effects on hemodynamic stability must be carefully evaluated; (2) in patients with a history of malignancy, particularly medullary thyroid carcinoma, the potential proliferative risks associated with GLP-1RAs must be weighed against their cardiovascular benefits; and (3) in frail elderly populations, dosing strategies should be optimized to avoid excessive weight loss and associated muscle wasting. In the future, biomarker-guided precision treatment approaches should be established—for example, using dynamic NT-proBNP monitoring combined with myocardial strain echocardiography to identify subgroups more likely to respond favorably to GLP-1RAs therapy.

In the future, large-scale clinical trials should be conducted to further clarify the molecular targets and specific pathways of GLP-1RAs. This would allow for the classification of drugs based on their efficacy across different pathways and optimize their use for individual conditions. Furthermore, exploring potential synergistic effects between different GLP-1RAs types could enhance their therapeutic benefits. With a clearer understanding of the mechanisms involved, more comprehensive experiments should be conducted to further elucidate the signaling pathways, minimize adverse effects, and provide better guidance for clinical drug application.

In conclusion, GLP-1RAs have shown significant promise in improving the prevention of cardiomyopathy in patients. However, the range of drugs that have been confirmed for use is still limited. Expanding the list of applicable drugs will be critical for achieving better preventive effects for both existing and emerging types of cardiomyopathy. Given these diseases’ high prevalence and diverse underlying mechanisms, the continued use of GLP-1RAs is supported. Research at various levels (such as cellular, molecular, and genetic) offers a new path to clarifying the mechanisms of action that are yet to be fully understood. Although the preventive and therapeutic effects of GLP-1RAs in cardiomyopathy have been initially elucidated, further research is necessary to refine the specific mechanisms of action and target pathways, ultimately expanding their clinical applications. Only through the seamless integration of in-depth mechanistic research and clinically contextualized applications can the translational leap of GLP-1RAs from bench to bedside be truly achieved.

## Abbreviations

T2DM, type 2 diabetes mellitus; CVDs, cardiovascular diseases; GLP-1RAs, glucagon-like peptide-1 receptor agonists; ESC, European Society of Cardiology; GLP-1, glucagon-like peptide-1; DPP-4, dipeptidyl peptidase-4; GLP-1R, glucagon-like peptide-1 receptor; ERS, endoplasmic reticulum stress; cAMP, cyclic AMP; PKA, protein kinase A; PI3K, phosphoinositide 3-kinase; MAPK, mitogen-activated protein kinase; ANG II, angiotensin II; T1DM, type 1 diabetes mellitus; DMH, dorsomedial hypothalamus; SGLT2is, sodium-glucose cotransporter 2 inhibitors; DCM, dilated cardiomyopathy; DC, diabetic cardiomyopathy; I/R, ischemia/reperfusion; EAT, Epicardial adipose tissue; HF, heart failure; OHP2, oral hypoglycemic peptide 2; Cx43, connexin 43; HG, high glucose; UTMD, ultrasound-targeted microbubble destruction; DXR, doxorubicin; TTS, Takotsubo syndrome; MACE, major adverse cardiovascular events.
